# A Late Bronze Age foreign elite? Investigating mobility patterns at Seddin, Germany

**DOI:** 10.1371/journal.pone.0330390

**Published:** 2025-09-10

**Authors:** Anja B. Frank, Jens May, Serena Sabatini, Franz Schopper, Robert Frei, Flemming Kaul, Susanne Storch, Svend Hansen, Kristian Kristiansen, Karin M. Frei

**Affiliations:** 1 Department of Earth System Science, Universität Hamburg, Hamburg, Germany; 2 Department of Research, Collections and Conservation, Environmental Archaeology and Materials Science, National Museum of Denmark, Kongens Lyngby, Denmark; 3 Brandenburgisches Landesamt für Denkmalpflege und Archäologisches Landesmuseum (BLDAM), Zossen, Germany; 4 Department of Historical Studies, University of Gothenburg, Gothenburg, Sweden; 5 Department of Geosciences and Natural Resource Management, University of Copenhagen, Copenhagen, Denmark; 6 Department of Prehistory, Middle Ages and Renaissance, National Museum of Denmark, Copenhagen, Denmark; 7 Anthropologische Bestimmungen und Projektbetreuung (ABP), Berlin, Germany; 8 Deutsches Archäologisches Instititut (DAI), Berlin, Germany; 9 Globe Institute, Lundbeck Foundation GeoGenetics Centre, Copenhagen, Denmark; University of Padova: Universita degli Studi di Padova, ITALY

## Abstract

During the Late Bronze Age (ca. 11^th^-8^th^ century BCE), far-reaching and extensive trade and exchange networks linked communities across Europe. The area around Seddin in north-western Brandenburg, Germany, has long been considered as at the core of one such networks. The degree of which the exchange practices involved in the circulations of goods and ideas was facilitated by people of different origins settling along the networks remains to be understood. To address this question, this study presents Sr isotope data of 29 cremated petrous bones from five neighbouring Late Bronze Age burial sites around Seddin, including the 9^th^ century BCE *Wickbold I* burial mound. Modern environmental samples and archaeological soil samples were also analysed for ^87^Sr/^86^Sr to establish a bioavailable reference baseline for the region. The results suggest that modern water and archaeological soil samples appear to be best suited proxies for defining a ^87^Sr/^86^Sr baseline that can reliably be used to trace Bronze Age mobility at Seddin, while the modern soil and plant sample ^87^Sr/^86^Sr data seem to reflect changes inherent to natural carbonate leaching of the glaciogenic surface sediments over time and/or recent anthropogenic contamination, such as fertilizers, rendering their use as representative archives for bioavailable Sr in the study of past human mobility, at least in the greater Seddin region, problematic. The comparison of the petrous bone ^87^Sr/^86^Sr signatures to the proposed water Sr isotope baseline reveals an overwhelming presence of non-locals in the investigated grave sites, with only two of 22 individuals falling within the local baseline. This study suggests complex mobility patterns of the elite community around Seddin during the Late Bronze Age.

## Introduction

**Fig 1:**
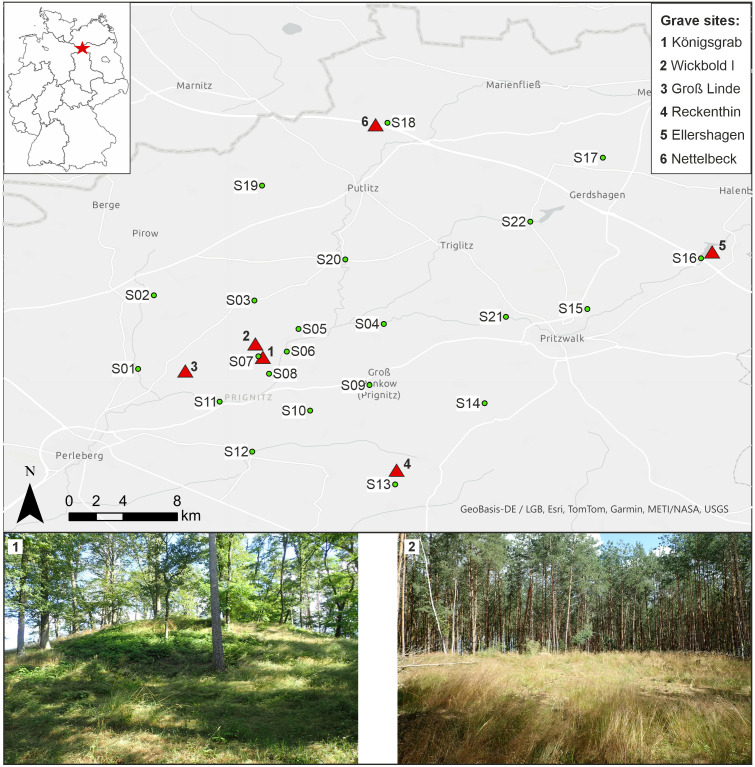
Overview map of the study area. Position of the investigated Bronze Age burials are indicated by red triangles (1-6) and baseline sampling sites are shown as green dots.

The abundance of well-preserved archaeological finds dating to Nordic Late Bronze Age (LBA) Periods IV-V (ca. 11^th^-8^th^ century BCE) suggests that by that time, the Prignitz in north-western Brandenburg, Germany, had developed into an important political and cultural area [1]. A large number of burial mounds, several housing graves with swords as well as impressive bronze and clay grave goods, in addition to documented settlements act as testimonials of the importance of the region [1–3]. The most prominent monument is the so called *Königsgrab* (the King’s grave) of Seddin (Fig 1), a 9^th^ century BCE monumental burial mound covering a remarkable polygonal stone chamber tomb rich in bronze grave goods [1,4–9]. Approximately 1 km north of the *Königsgrab* lies another burial mound, the so-called *Wickbold I*. Just like the *Königsgrab*, *Wickbold I* was also erected during the 9^th^ century BCE and furnished with rich bronze grave goods (Supplement; [1,10,11]); but unlike the *Königsgrab* little has been published on *Wickbold I*. The grave goods from those graves show connections to foreign traditions, with most suggesting an integration of Seddin into the culture of the Nordic Bronze Age. For example, a knife from *Wickbold I* shows parallels to grave goods from Denmark and its socked bronze axes belong to a group of small, but variable axes found in Sweden, Denmark, northern Germany and even parts of the Netherlands [12]. At the same time, a completely foreign object found in Seddin is the phalera that was used to cover the urn of the *Königsgrab*, as such bronzes are almost exclusively found in the urnfield culture of southern Europe [13]. While the bronze amphora from the *Königsgrab* was fashioned in a non-local style exhibiting parallels with similar items from the prominent Etruscan town of Veii in central-western Italy [2], similar vases were also found close to Rorbæk on the Jutland Peninsula [14–16], in Herzberg, Brandenburg, and in Unia in western Poland, supporting also a northern distribution [17]. Such rich grave goods as well as the use of bronze urns and the construction of monumental grave mounds, which are typical for the funerary practice of the European elite at the time [2,18], strongly suggest that the Seddin area was likely an important and central node within the long-distance network connecting northern and southern, potentially even eastern and western, Europe.

It is likely that this far-reaching trade and exchange practice demanded extensive human mobility. However, to date, no mobility studies have been conducted on human remains from the LBA Prignitz. Indeed, a considerable part of the archaeological evidence was excavated between the late 19^th^ and early 20^th^ century [e.g., 14], when provenancing of human remains was not yet possible. Furthermore, the cremated human remains characteristic for this LBA period, were generally not considered or kept. Accordingly, the human remains from the *Königsgrab* went missing. Recent excavations carried out between 2016 and 2018 at *Wickbold I* unearthed cremated human remains, thus, representing a unique opportunity to investigate human mobility in the Seddin area. Furthermore, it has been shown that the otic capsule within petrous bones, preserves the original ^87^Sr/^86^Sr signature even under high temperatures and cremation and can, thus, be used to study human mobility [19,20]. As the formation of the petrous bone concludes at the age of two without remodelling later in life, its Sr isotope composition reflects an individual’s dietary intake up to that age [20] and can be used to understand whether an individual spent the first years of their childhood away from the area they were buried. Cremated petrous bones suitable for Sr isotope investigations from *Wickbold I* together with petrous bones from four roughly contemporary neighbouring burial contexts were thus chosen to investigate LBA mobility around Seddin.

The successful application of ^87^Sr/^86^Sr isotope investigations to trace an individual’s childhood origin hinges on the availability of a bioavailable ^87^Sr/^86^Sr reference baseline of the area the remains were unearthed from. In recent years such reference baselines have been developed for many regions and even countries in Europe [21–29], but no suitable bioavailable ^87^Sr/^86^Sr reference data exists for the Prignitz. As to date, no consensus exists on how to establish relevant and appropriate bioavailable ^87^Sr/^86^Sr reference baselines for archaeological mobility studies, and different proxy materials are used. These proxies range from archaeological human skeletal remains [30–32], over modern and archaeological faunal skeletal remains [25,33,34] to modern environmental samples [21,22,24,27,30]. The suitability of these different proxies is still being evaluated as none of them are a perfect representation of dietary ^87^Sr/^86^Sr available to ancient humans and seem to be region dependent [30,35–37]. Considering the above-mentioned baseline issues, we opted for a multi-proxy environmental approach to interpret the Sr isotope data of the petrous bones sampled in this study. We present ^87^Sr/^86^Sr data from environmental samples (plants, soils and surface waters) taken from 22 sites around Seddin and from the cultural layer (soil) underneath and inside of the *Königsgrab* as reference material. Additionally, ^14^C dating and anthropological investigations were conducted on the human remains analysed herein. The results provide first insights into mobility and childhood origin of the LBA population of the Prignitz.

## Background

### Study area

**Fig 2:**
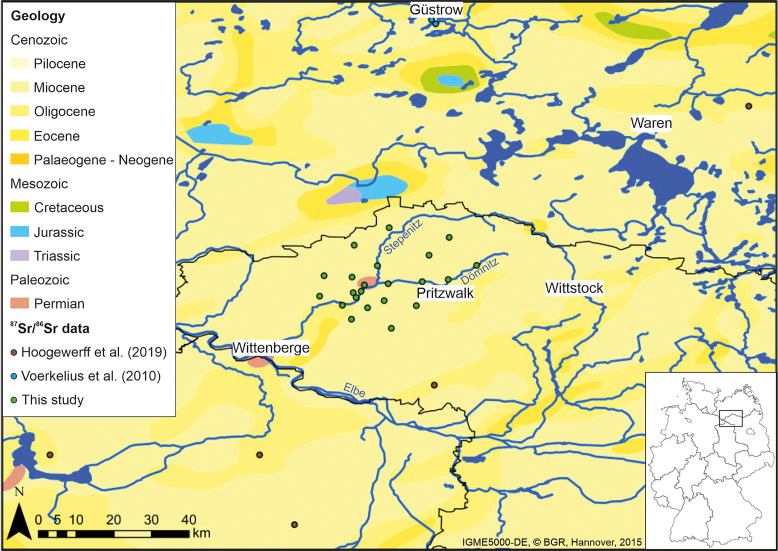
Surface lithological map of the study area [38]. The dots mark sites where environmental samples were taken to study the area’s bioavailable Sr isotope baseline.

The Prignitz is the north-western most region of the state of Brandenburg, and borders the German states of Mecklenburg-Vorpommern to the north, Lower Saxony to the west and Saxony-Anhalt to the south. Its south-western border is marked by the river Elbe (Fig 2), which continues on to the North Sea granting the area a riverine connection to the coast. The river Stepenitz and its tributary, the Dömnitz, further shape the landscape merging just west of Pritzwalk and into the Elbe by Wittenberge.

The Prignitz is characterised by a flat topography with rolling plains disrupted by river valleys shaped by glacial processes, with the last glaciation dating back to the younger Saalian [8]. The resulting sediments are very homogeneous and dominated by Cenozoic glacial to fluvial-glacial deposits (Fig 2) [38]. Soil formation started in the Late Glacial [39,40]. Typical soils are Fahlerde, Braunerde and Gleysols consisting of sand, silt, clay and marl [38], with variable contents of organic material. Today the landscape is dominated by agricultural fields, which are disrupted by forests and settlements.

### Archaeological context

Many archaeological discoveries dating to the 11^th^ – 8^th^ century BCE have been made along the river Stepenitz with a particular dense cluster found around the modern settlement of Seddin. The most prominent monument is the so called *Königsgrab*, one of the largest and richest period V burial mounds in all of Northern Central Europe. Its polygonal stone burial chamber was furnished with over 40 ceramic and metal items including a full-hilted bronze sword and a magnificent bronze biconical vessel [41]. The richness of grave goods indicates Seddin’s role as a centre of influence and wealth during the LBA [42,43] and suggest that the individual(s) buried in the *Königsgrab* probably held a key role in the North-South trade and exchange networks across Europe [3,13,15,43–46]. It has been estimated that the local elite could have inhabited an area of ca. 100km^2^ encompassing over 200 archaeological sites, many of which are burial mounds, and that their political and military influence likely reached even further [1].

While the importance and wealth of the Seddin elite has generally been established on the base of the richness of the *Königsgrab*, they are also reflected in the less well-known and studied *Wickbold I* burial mound. *Wickbold I* was discovered in 1888, a decade before the *Königsgrab*, in the forest *Wickboldsches Wäldchen*, about 1 km north of the *Königsgrab*. The burial mound of *Wickbold I* consists of a polygonal stone burial chamber equipped with rich bronze grave goods [47], including a sword, an axe, a knife, and tweezers (S1 Fig). A door urn served as the ossuary and likely distinguished an individual(s) of high importance [11]. *Wickbold I* marks the centre of a burial ground and includes, besides the main burial chamber, also graves older than Period V and is surrounded by Period V and VI burial mounds and flat graves [10]. The burial chamber of *Wickbold I* was removed at the time of the discovery in 1888 and the mound was removed without documentation. Between 2016 and 2018, an area of the *Wickbold I* burial mound was re-excavated uncovering the remains of several cremation burials (S2 and S3 Figs). The findings of this recent excavation have yet to be published in its entirety, but a preliminary report is given here in supplement S4 File.

## Methods

### Material

Cremated remains from the *Wickbold I* excavation and from four other neighbouring sites were investigated (Fig 1, Table 1, S4 File). For Sr isotope analysis, the petrous portion was sampled, which fully crystallises by the age of two with no remodelling thereafter. Three almost complete and six fragmented petrous bones from *Wickbold I* are the main focus of this study. Additionally, 20 petrous bones from the roughly contemporary graves of *Groß Linde, Reckenthin, Ellershagen,* and *Nettelbeck* have also been included to provide a more complete picture of ancient mobility in the Prignitz during the Nordic LBA.

**Table 1:** Overview of the investigated burials as well as petrous bones and archaeological soils including anthropological information, [Sr] and ^87^Sr/^86^Sr signatures.

Excavation site	Burial	Period	Sample	ID	Material	Anthropology	Sr (ppm)	^87^Sr/^86^Sr	2SE
Wickbold I	Complex A – destroyed cremation burials	Probaly Period II – V	KF2093	Fundkonzentration2-SK2017:307/27/5	Petrous bone -right	6 individuals, three probably male and adult, one infans I	74.58	0.71437	0.00002
			KF2094	Fundkonzentration2-SK2017:307/29/4	Petrous bone -right		90.10	0.71369	0.00002
			KF2095	Fundkonzentration15-SK2018:324/Tuete38	Petrous bone- left		69.41	0.71388	0.00002
			KF2099-A	Streufunde_Sk2018:324/Tuete51	Petrous bone- fragment		79.63	0.71447	0.00001
			KF2099-B	Streufunde_Sk2018:324/Tuete51	Petrous bone- fragment		101.71	0.71197	0.00001
			KF2099-C	Streufunde_Sk2018:324/Tuete51	Petrous bone- fragment		99.70	0.71088	0.00001
	FC14 – destroyed cremation burial		KF2096	Fundkonzentration14-SK2018:324/Tuete33	Petrous bone -fragment	1 individual, late juvenile – early adult	59.00	0.71355	0.00001
	Grave S4 - destroyed urn burial (partially excavated)	Probably Period III-IV	KF2097*	Befund4-Sk2018:324/Tuete2	Petrous bone- fragment	1 individual, juvenile – senile	61.25	0.71418	0.00001
			KF2098*	Befund4-Sk2018:324/Tuete3	Petrous bone- fragment		61.10	0.71411	0.00001
Reckenthin	Grave 1 – single urn burial (potentially primary burial)	Probably Period V	KF2373	1971-20-1-2	Petrous bone- left	1 individual, probably female and late juvenile – senile	73.67	0.71267	0.00002
	Grave 2 – cremation burial in a pit (inside stone ring)	Period IV	KF2374-A	1971-20-2-2	Petrous bone -right	2 individuals, one infans II – juvenile, one infans I	53.62	0.71369	0.00001
			KF2374-B	1971-20-2-2	Petrous bone -right		70.88	0.71468	0.00002
	Grave 4 – single urn burial	Period IV	KF2375	1971-20-4-2	Petrous bone- left	1 individual, late juvenile – adult	47.13	0.71373	0.00002
	Grave 7 – separate urn burials	Probably Period IV	KF2376-A	1971-20-7-2	Petrous bone -right	2 individuals, one probably female and late juvenile – adult, one infans I	22.49	0.71409	0.00002
			KF2376-B	1971-20-7-2	Petrous bone- left		55.12	0.71419	0.00002
Groß Linde	Single urn burial	Period V or VI	KF2377	2006:464_3	Petrous bone -right	1 individual, infans I	56.68	0.71414	0.00003
Nettelbeck	Polygonal grave chamber of grave mound 2 – separate urn burials with some scattering of remains outside the urns	Period V – VI	KF2378-A*	1979-5-2-6	Petrous bone -right	1 individual, probably female and adult – senile	57.01	0.71382	0.00002
			KF2378-B*	1979-5-2-6	Petrous bone- left		61.80	0.71344	0.00002
	Polygonal grave chamber of grave mound 3 – separate urn burial	Period V	KF2379-A*	1979-5-3-5	Petrous bone -left	1 individual, probably male and adult – mature	58.68	0.71383	0.00003
			KF2379-B*	1979-5-3-5	Petrous bone -right		56.52	0.71391	0.00002
	Polygonal grave chamber of grave mound 4 – separate urn burial with some scattering of remains outside the urns	Period V	KF2380-A*	1979-5-4-5	Petrous bone -right	2 individuals, one probably female and late juvenile – senile, one infans I – infans II	56.73	0.71303	0.00002
			KF2380-B*	1979-5-4-5	Petrous bone- left		60.47	0.71273	0.00001
			KF2380-C1	1979-5-4-5	Petrous bone- fragment		163.96	0.71412	0.00001
			KF2380-C2	1979-5-4-5	Petrous bone- fragment		77.37	0.71267	0.00002
Ellershagen	Grave 1 – separate urn burial	Period III	KF2381	1985-8-1-2	Petrous bone -right	2 individuals, one probably female and late juvenile – senile, one infans I	73.91	0.71474	0.00002
	Grave 2 – single urn burial	Period III	KF2382	1985_2_2	Petrous bone -right	1 individual, probably male and adult – early mature	70.56	0.71437	0.00002
	Grave 5 – single urn burial with some scattering of remains outside the urn (primary grave with stone ring)	Period IV	KF2383-A*	1985-8-5-2	Petrous bone -right	1 individual, probably female and late adult – mature	61.83	0.71384	0.00001
			KF2383-B*	1985-8-5-2	Petrous bone- left		66.07	0.71394	0.00002
	Grave 6 – single cremation burial in pit (post burial)	Pre-roman iron age	KF2384	1985-8-8-1-1	Petrous bone- left	1 individual, probably female and juvenile – adult	76.03	0.71137	0.00003
Königsgrab			KMF111	PRH2001:188/Grabkammer	soil – grave chamber	–	0.36	0.71023	0.00002
			KMF112	PRH2003:130/Schnitt2003	soil – cultural layer	–	0.04	0.71025	0.00006
			KMF113	PRH2013:63/Schnitt1	soil – cultural layer	–	0.07	0.71077	0.00004
			KMF114	PRH2013:63/Schnitt2	soil – cultural layer	–	0.05	0.71124	0.00003
			KMF115	PRH2017:86/Schnitt12	soil – cultural layer	–	0.03	0.71057	0.00002

*Samples from individuals represented by multiple petrous bones.

To create ^87^Sr/^86^Sr reference baselines for interpreting the human ^87^Sr/^86^Sr results, we further took various environmental samples. Modern day water, plants and soils were taken at 22 locations within the immediate surroundings of the investigated excavation sites (Fig 1, Table 2). The sites were located, where possible, in forested areas. At each site three plant and soil samples were taken within a ~ 150m radius to determine the site’s average bioavailable Sr isotope composition. The soils were sampled from the topsoil, ~ 10 cm below the moor layer. Leaves from shrubs or small trees up to 2.5m high were sampled from different heights of the plant and combined to yield a representative and homogenised plant sample. The sampled plant species depended on the availability at the site and included inter alia beech, blackberry and maple. The water samples were taken from rivers, streams and drainage channels and, where possible, in close proximity to the other environmental proxies. Finally, five soil samples were taken from the cultural layers of the *Königsgrab*, which is suggested to be roughly contemporaneous to the *Wickbold I* site and only ~1 km away (Fig 1). One sample was taken from the clay floor of the grave chamber (KMF111) and four from the cultural layers underneath the burial mound (Table 1).

**Table 2:** Geographic location, [Sr] and ^87^Sr/^86^Sr signatures of modern environmental (soil, plant and surface water) samples taken for the baseline.

			Soil leachates			Plants			Surface water			
Location	Latitude	Longitude	Sr (ppm)	^87^Sr/^86^Sr	2SE	Sr (ppm)	^87^Sr/^86^Sr	2SE	Sr (ppm)	^87^Sr/^86^Sr	2SE	Water body
S01	53.12923	11.89322	2.58	0.70875	0.00003	35.91	0.70886	0.00002	0.21	0.71006	0.00001	Schlatbach
S02	53.17817	11.90373	8.01	0.70963	0.00002	18.46	0.71021	0.00002	0.28	0.71001	0.00001	Schlatbach
S03*	53.17471	11.97058	2.77	0.70929	0.00002	14.33	0.70969	0.00002	0.54	0.70756	0.00002	Drainage
S04	53.15913	12.05664	1.23	0.71073	0.00003	10.85	0.71086	0.00001	0.26	0.71117	0.00001	Tributary of Doemitz
S05	53.15579	11.9999	3.25	0.70873	0.00002	18.46	0.70906	0.00002	0.22	0.70906	0.00002	Tributary of Stepenitz
S06	53.14077	11.99211	2.69	0.70883	0.00002	24.81	0.70919	0.00002	0.23	0.71035	0.00003	Stepenitz
S07*	53.13763	11.97322	3.10	0.70699	0.00002	41.19	0.70607	0.00003	0.17	0.71024	0.00003	Tributary of Stepenitz
									0.34	0.70853	0.00002	Drainage
S08	53.12603	11.98029	0.36	0.71263	0.00003	12.28	0.71088	0.00002	0.20	0.70957	0.00003	Tributary of Stepenitz
S09	53.11853	12.0471	3.80	0.70852	0.00002	30.97	0.70889	0.00002	0.25	0.71040	0.00003	Panke
S10	53.10152	12.00764	8.01	0.70866	0.00002	19.91	0.70906	0.00002	0.21	0.70989	0.00003	Retziner Muehlbach
S11	53.10749	11.94744	3.13	0.71048	0.00002	20.26	0.71058	0.00001	0.22	0.71022	0.00003	Stepenitz
S12	53.07427	11.96912	3.85	0.70905	0.00001	8.83	0.71646	0.00001	0.34	0.70917	0.00001	Jeetzbach
S13	53.05246	12.06419	0.22	0.71106	0.00002	17.30	0.71331	0.00001				
S14	53.10643	12.12368	5.51	0.70648	0.00002	86.14	0.70670	0.00001	0.25	0.71032	0.00002	Panke
S15*	53.16906	12.19199	1.36	0.71257	0.00002	15.96	0.71211	0.00001	0.21	0.71112	0.00001	Tributary of Doemitz
S16	53.20279	12.26759	0.38	0.71184	0.00002	26.42	0.71162	0.00001	0.30	0.71022	0.00001	Doemniz
S17	53.26967	12.20232	0.29	0.71272	0.00002	7.53	0.71251	0.00002				
S18	53.29287	12.05906	0.95	0.70755	0.00002	29.46	0.70978	0.00001	0.20	0.71015	0.00001	Sabel
S19	53.2511	11.97566	1.18	0.70710	0.00002	38.11	0.70897	0.00001	0.25	0.71017	0.00002	Tributary of Sagast
S20	53.20201	12.03103	3.74	0.71346	0.00002	11.41	0.71316	0.00003	0.31	0.71071	0.00002	Freundenbach
S21	53.16386	12.13787	7.45	0.70502	0.00003	59.60	0.70497	0.00002	0.25	0.71035	0.00002	Elsbaek
S22	53.22713	12.15407	4.53	0.71254	0.00001	30.41	0.71301	0.00002	0.38	0.71012	0.00002	Kuemmernitz

*Water sample was not taken in the immediate vicinity of the soil and plant samples, but up to 1 km from the given GPS coordinate.

The 2016–2018 excavation at *Wickbold I* uncovered an archaeologically compromised and complex situation also regarding the chronological order of the human remains. Therefore, to better understand the chronological sequence of the various find concentrations (FC), cremated remains and charcoal samples were selected for ^14^C dating (S3 Fig). Unfortunately, it was not possible to ^14^C date the petrous bone samples themselves as they were heavily mineralised, and not enough organics were preserved to date them directly. Therefore, nine other cremated limb bones and five charcoals were selected instead (Table 3).

**Table 3:** Carbon dating results of the Wickbold I long bone and charcoal samples.

Sample	ID	Description	FC	fraction Modern	±	D14C (‰)	±	14C age (BP)	±	2σ cal.BC (95.4%)
246559	2017:307/24/3*	1 Long bone, compacta, under limb	1	0.7099	0.0014	−290.1	1.4	2750	20	967−827
245884		1 Long bone, compacta, under limb		0.7077	0.0068	−292.3	6.8	2780	80	1190−801
246560	2017:307/28/5*	1 Long bone, compacta, under limb	1	0.7070	0.0013	−293.0	1.3	2785	15	1005−856
245885		1 Long bone, compacta, under limb		0.7162	0.0083	−283.8	8.3	2680	100	1118−542
245886	2017:307/22/3	1 Long bone, compacta, under limb	2	0.8329	0.0115	−167.1	11.5	1470	120	260-824AD
245887	2017:307/27/5	1 Long bone, compacta, tibia?	2	0.6996	0.0046	−300.4	4.6	2870	60	1256−899
246561	2017:307/27/5*	1 Long bone, compacta	2	0.6992	0.0013	−300.8	1.3	2875	20	1124−939
245888		1 Long bone, compacta		0.6781	0.0014	−321.9	1.4	3120	20	1441−1304
245889	2017:307/30/3	1 Long bone, compacta, under limb, adult	2	0.7187	0.0087	−281.3	8.7	2650	100	1050−478
246562	2018:324/Tüte10	1 Long bone, compacta, upper limb	2	0.6825	0.0013	−317.5	1.3	3070	20	1407−1271
246563	2018:324_Tüte37*	1 Long bone, compacta	10	0.6971	0.0013	−302.9	1.3	2900	20	1191−1012
245890		1 Long bone, compacta		0.7146	0.0093	−285.4	9.3	2700	110	1199−543
246564	2018:324_Tüte33	1 Long bone, compacta	14	0.6962	0.0013	−303.8	1.3	2910	15	1198−1016
245873	2017:307	Pine tree (charcoal)	2	0.6957	0.0014	−304.3	1.4	2915	20	1206−1017
245874	2018:324	Oak tree (charcoal)	7	0.4154	0.0010	−584.6	1.0	7060	20	6009−5888
245875	2018:324	Pine tree (charcoal)	8	0.6871	0.0014	−312.9	1.4	3015	20	1382−1134
245876	2018:324	Oak tree (charcoal)	10	0.6853	0.0014	−314.7	1.4	3035	20	1387−1220
245877	2018:324	Oak tree (charcoal)	13	0.6801	0.0013	−319.9	1.3	3095	20	1422−1292

*Long bone samples that produced a residue, which was dated as well*.*

All the archaeological material (human bone, soil from the cultural layer of the *Königsgrab* and charcoal) included in this study were provided by Brandenburgisches Landesamt für Denkmalpflege und Archäologisches Landesmuseum (BLDAM), whose director is one of the co-authors, and the archive IDs are listed in Tables 1 and 3. This study was supported by the BLDAM and the Deutsches Archäologisches Instititut (DAI), who consented for the study to take place. No permits were required for the sampling of modern environmental samples and the study complied with all relevant regulations.

### Osteology

The complete cremated remains of the investigated archaeological sites were inspected visually to determine an estimate of the number of individuals. Hence, anthropological investigations also considered recovered human remains without any associated petrous bones. The approximate age and sex of the cremated individuals was determined based on morphological-morphognostic criteria [48–53]. A brief overview of the findings is given in Table 1. For burials with multiple individuals, the determined age and sex could commonly not be linked to the petrous bones analysed for Sr. Hence, the anthropological information listed in Table 1 is only provided for a better understanding of the archaeological contexts.

### ^14^C dating

The ^14^C analysis were performed by the Keck Carbon Cycle AMS radiocarbon measurement facility of the University of California, Irvine (UCI). Unfortunately, the cremated bone remains turned out to have been burned sufficiently enough to destroy the collagen, but temperatures were not high enough for the bones to become calcinated, allowing for the formation of dateable carbonate crystals. The crystallinity index [54,55] was not measured, as it was obvious that there was not enough dateable carbonate crystal for most samples. Due to the uniqueness of the contexts, analyses were performed anyway and all results have been corrected for isotopic fractionation according to the conventions of [56], with δ^14^C values measured on prepared graphite using the AMS spectrometer. Samples 246559–246564 were leached overnight under vacuum with 1N acetic acid prior to hydrolysis with 85% phosphoric acid. Samples 245873 and 245884 were treated with acid-base-acid (1N HCl and 1N NaOH, 75°C) prior to combustion. Several bone samples produced a black powder residue when treated with HCl, and this material was treated as bone charcoal. This black material might have been refractory soil carbon rather than bone carbon from the sample and, thus, the obtained dates might show the age of the soil rather than that of the human cremated remains. For the above-mentioned reasons, the results of the ^14^C dating from the Wickbold I (Table 3) presented in this study need to be considered with great caution.

### Sr isotopes

All petrous bone samples were investigated for their Sr concentration and isotope composition, regardless of whether two were suggested to be from one individual, to test their reproducibility and potentially constrain the number of investigated individuals further. The sampling of the cremated petrous bones for Sr isotope analysis was done following the methods described by [19,57] and 1–2 mg of the densest part of the otic capsule was sampled. The samples were prepared and collected using a dental diamond drill, dissolved in pre-cleaned Teflon beakers using a 1:1 solution of 0.5 ml 6M HCl and 0.5 ml 30% H_2_O_2_, spiked with a ^84^Sr enriched tracer to determine the Sr concentration via isotope dilution (ID) and then dried down on a hot plate at 100°C.

The archaeological and modern soils were leached following the extraction procedure applied in the Europe-wide soil-based bioavailable strontium isotope survey, which was performed as part of the Geochemical Mapping of Agricultural and Grazing Land Soil (GEMAS) framework program [58]. For this 1g of air-dried soil was reacted with 5 ml of a 1M ammonium nitrate (NH_4_NO_3_) solution in an overhead shaker for 2h. While the 1g of soil constituted one soil sample for the archaeological soils, a composite sample consisting of 0.33g soil from each of the three samples taken at one site was used for the modern soils. After centrifuging, 1 ml aliquots were pipetted into pre-cleaned Teflon beakers and spiked. Finally, the samples were dried down on a hot plate at 100°C.

To remove any dust, the plant samples were cleaned using ultra-clean water (Milli Q (MQ) system) and subsequently air-dried. A composite sample consisting of 0.033g of each plant sample taken at one site was weighed into a pre-cleaned ceramic crucible to achieve a final sample weight of 0.1g. The samples were incinerated in a laboratory muffle furnace at 750°C for 5h before being transferred into pre-cleaned Teflon beakers using ~2 ml MQ. A ^84^Sr enriched tracer was added to the samples, before they were dried down on a hot plate at 100°C. To achieve complete digestion and sample-spike homogenisation, the samples were subsequently reacted with 1 ml concentrated HNO_3_, and then, dried down again.

The surface water samples were filtered using 0.45µm syringe filters, before pipetting 10 ml aliquots into pre-cleaned Teflon beakers. The samples were spiked with a ^84^Sr-enriched tracer and dried down on a hot plate at 100°C overnight.

Sr column separation and thermal ionisation mass spectrometry (TIMS) measurements were performed at the Department of Geoscience and Natural Resource Management (IGN) at the University of Copenhagen and followed largely the methods used by [25]. 1 ml pipette tips were fitted with pre-cleaned, pressed-in filters to serve as disposable extraction columns. The columns were charged using 200 µl pre-cleaned SrSpec™ resin (50–100 mesh; Eichrome Inc./Tristchem) conditioned with 3M HNO_3_. The prepared samples were re-dissolved in a few drops of 3M HNO_3_, loaded onto the columns and washed using ~10 ml of 3M HNO_3_. Finally, the Sr was collected using 2 ml of MQ and dried down on a hot plate at 120°.

A VG Sector 54 IT mass spectrometer equipped with eight Faraday detectors was used to determine the Sr concentrations and isotope compositions. The samples were loaded on previously outgassed 99.98% single Re filaments using 2.5µl of a Ta_2_O_5_-H_3_PO_4_-HF activator solution. The analyses ran in a dynamic multi-collection mode at analysing intensities ≥ 1V for ^88^Sr and temperatures between 1300–1350 °C. Runs of the standard reference material SRM 987 returned an average ^87^Sr/^86^Sr ratio of 0.710239 ± 0.00002 (n = 8, 2σ), which is slightly offset to the mean SRM 987 value of 0.710245 published by [59]. The measured ^87^Sr/^86^Sr values of the samples were corrected accordingly. The within-run precessions (2SE) of the individual runs were up to 0.00006, but generally ≤ 0.00003 (Tables 1 and 2). Procedural Sr blanks were in the order of 20pg to 60pg of strontium with blank ^87^Sr/^86^Sr signatures between 0.7095 and 0.7122. However, no blank correction was conducted as the blank contribution is considered insignificant compared to the overall sample Sr of typically ≥200ng.

## Results

### ^14^C dating

As mentioned above, the radiocarbon dates of the *Wickbold I* samples should be interpreted with great caution (Table 3). The analytical error of some of the dating is very high and in three cases (245885, 245889, 245890) the obtained calibrated time span is so wide that they cannot possibly provide any useful information about the FC they come from. One sample (245886) appears to be from the first millennium CE and suggests the presence of later historical depositions in the area. One oak charcoal was dated to the beginning of the 6^th^ millennium BCE (245874) and is therefore not considered any further. The results from the remaining samples were combined to date the different FC (S3 Fig). Considerable discrepancies appeared between the dates obtained from bones and those from charcoal in FC 2 and FC 10. As it is not possible to understand whether the bone charcoal was from the bones or from the refractory soil, it is difficult to discuss the nature of the discrepancy. Based on the obtained dates, it appears that one sample from FC 2 (246562) represents an Early Bronze Age (Nordic Period II-III) grave predating the construction of the *Wickbold I* mound. Further, samples 246564, 246563 and 246561 also suggest that depositions from the end of the Early Bronze Age and the beginning of the Late Bronze Age (Period III-IV) might have been present prior to the construction of the mound. The dates obtained from the sampled bones in FC 1 align with the general Late Bronze Age (Period IV-V) of the *Wickbold I* burial ground and are likely contemporary to the construction of the mound.

### Osteological investigations

A preliminary investigation on *Wickbold I* identified two right-sided and one left-sided petrous bone as well as six petrous bone fragments [60]. During excavation, the three almost complete petrous bones (KF2093-KF2095) and the three possible petrous bone fragments (KF2099-A – KF2099-C) were found together with additional cremated bone pieces used for the anthropological investigations within one confined complex (complex A, S4 File). Based on the complete recovered bone assemblage, it was suggested to hold at least five individuals. Three of the proposed five individuals were determined to be adults and potentially male, while the other two were subadult and of indeterminable sex. KF2093 was linked to the cremated remains of the male adults, while KF2095 was associated with the remains of one male adult as well as another adult individual of smaller statue. For KF2094 and the three fragments KF2099-A – KF2099-C it was also not possible to tell, whether these were from one of the adults or the subadults. The petrous bone fragments KF2096-KF2098 are from two additional adult individuals of undetermined sex and KF2097 and KF098 were determined to be from one individual as they belonged to a closed find.

The petrous bone sample from *Groß Linde* is right-sided and from an infans I of undetermined sex. The six samples from *Reckenthin* consist of three left and three right petrous bones. Even though the burials held six individuals, the petrous bones likely correspond only to five individuals. KF2373 is from one adult female, KF2375 from on adult of undetermined sex, KF2374-A from one subadult of undetermined sex and KF2374-B from another subadult of undetermined sex. KF2376-A and KF2376-B could be either from an adult female or an infant of undetermined sex. However, based on the morphology both are most likely from the infant. The *Ellershagen* graves included five, potentially six individuals. The investigated samples consisted of two left- and three right-sided petrous bones, which correspond to only four individuals, as KF2383-A and KF2383-B are suggested to come from the same adult female. KF2384 is from another adult female, while KF2381 and KF2382 could not be assigned to a specific individual, but each belonged to a close find. The petrous bones from *Nettelbeck* consist of three left- and three right-sided petrous bones as well as two fragments that could not be assigned to either side. The anthropological investigations suggested the presence of at least four individuals. KF2378-A and KF2378-B are from an adult female and KF2379-A and KF2379-B from an adult male. KF2380-A and KF2380-B as well as KF23380-C1 and KF2380-C2 are from a cremation of two individuals, one an adult female and one a subadult individual of indeterminable sex.

### Strontium results

**Fig 3:**
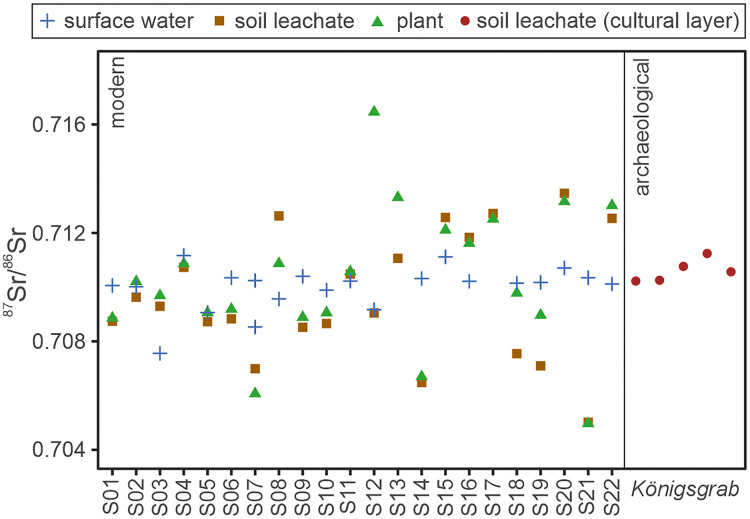
Bioavailable Sr isotope data of the environmental samples. ^87^Sr/^86^Sr data recorded for soil leachates (modern and archaeological), plants and surface waters around Seddin.

The modern environmental samples returned a wide range in ^87^Sr/^86^Sr values between 0.7050 and 0.7165 (Table 2, Fig 3). Both extreme values were measured in plant samples, which are characterised by an average ^87^Sr/^86^Sr value of 0.7103 ± 0.0026 (1σ, single standard deviation). The plant [Sr] varies between 7.5 ppm and 86 ppm. The soil leachates are also characterised by a wide range in ^87^Sr/^86^Sr values between 0.7050 and 0.7135, with an average ^87^Sr/^86^Sr value of 0.7097 ± 0.0023 (1σ). These ranges fit well with soil leachate ^87^Sr/^86^Sr values between 0.7050 and 0.7098 reported by [58] for similar glacial sediment outcrops in the larger vicinity of Seddin (Fig 2), but our soil leachate and plant ^87^Sr/^86^Sr ranges are extended towards more radiogenic signatures. The leached bioavailable soil [Sr] vary between 0.22 ppm and 8.0 ppm. The surface water ^87^Sr/^86^Sr signatures are more homogenous compared to the plant and soil leachate ^87^Sr/^86^Sr values, ranging from 0.7076 to 0.7117 with an average ^87^Sr/^86^Sr value of 0.7100 ± 0.0008 (1σ). Spring water ^87^Sr/^86^Sr values for drinking water sourced near Güstrow (0.7102–0.7103; [61]) are very similar to this average surface water ^87^Sr/^86^Sr value. The [Sr] of the investigated surface waters range between 0.17 ppm and 0.54 ppm.

The ^87^Sr/^86^Sr values and [Sr] of the archaeological soil samples are listed in Table 1 and plotted in Fig 3. The soil samples from the cultural layer of the *Königsgrab* returned ^87^Sr/^86^Sr values between 0.7102 and 0.7112 with an average value of 0.7106 ± 0.0004 (1σ). The leached bioavailable [Sr] of the archaeological soils vary between 0.20 ppm and 2.9 ppm. Both, the range in ^87^Sr/^86^Sr values and [Sr] measured for the cultural layer of the *Königsgrab* fall within the respective ^87^Sr/^86^Sr and [Sr] ranges of the modern environmental proxies.

The studied cremated human petrous bone samples are characterised by a range in ^87^Sr/^86^Sr values between 0.7109 and 0.7147, providing an average value of 0.7132 ± 0.0014 (1σ) (Table 1). The [Sr] vary between 22 ppm and 164 ppm. The ^87^Sr/^86^Sr values and [Sr] of petrous bone pairs suggested by the anthropological investigations to be from the same individuals are generally within <0.0004 and ~5% of each other, respectively. The left petrous bone KF2376-B and its right counterpart KF2376-A, however, returned a slightly higher difference in Sr concentration (~13%). Further, the four petrous bone samples from *Nettelbeck*, which could correspond to only two individuals (KF2380-A, KF2380-B, KF23380-C1 and KF2380-C2) did not return two distinguishable sets of Sr isotope signatures. While KF2380-B and KF2380-C2 have similar ^87^Sr/^86^Sr signatures and [Sr], KF2380-A has a lower [Sr] and KF2380-C1 has a significantly more radiogenic ^87^Sr/^86^Sr value and higher [Sr].

## Discussion

### Constraining a local bioavailable Sr isotope baseline

Bioavailable Sr, sourced from mineral weathering of soils and rocks and from exogenous deposition, is directly taken up by humans through their diet and incorporated within the skeleton [34,62]. As no significant fractionation occurs between the uptake of bioavailable ^87^Sr/^86^Sr and its skeletal incorporation [63], the comparison of skeletal ^87^Sr/^86^Sr ratios to the bioavailable ^87^Sr/^86^Sr range characterising the area the human remains were unearthed from can help us identify individuals with a non-local origin. This useful feature has resulted in increased efforts in defining adequate baselines, and bioavailable ^87^Sr/^86^Sr data are now available from many regions [21,22,24,26,28,58] and even countries [23,25,29,30,64–66]. However, Northern Germany, and in particular the North German Basin, had until very recently only a low coverage of bioavailable Sr isotope data. A recent study published new water-based Sr isotope data for Central and northeast Germany, but with special emphasis on mountainous regions such as the Erzgebirge/Fichtelgebirge and Harz Mountains [67]. The Prignitz part of the North German Basin, where the investigated individuals were unearthed, is still underrepresented in terms of baseline Sr isotope data.

**Fig 4:**
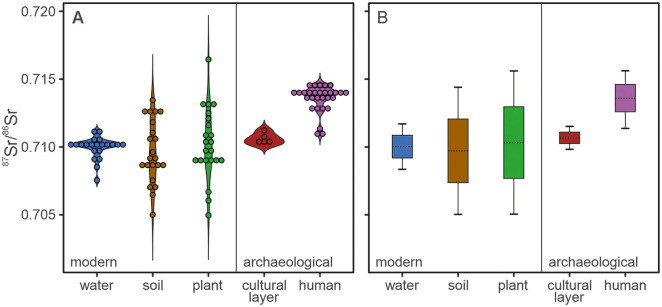
Bioavailable Sr data and calculated baselines for the study area. A) Violin plot displaying the spread in proxy ^87^Sr/^86^Sr signatures for water, modern soil leachate and plants as well as the cultural soil layer of the *Königsgrab* and investigated petrous bones. B) Calculated bioavailable Sr isotope baseline for the study area based on the different proxies. The dashed line represents the average value, the box the single and the whiskers the double standard deviation.

Furthermore, it is also debated which methods/proxy archives are the most suitable and adequate ones to construct reliable bioavailable Sr isotope baselines for tracing the mobility of ancient humans. For example, the geographical extents of past studies vary greatly, resulting in site-specific [68,69] to country-wide baselines [23,25] and the applied statistical methods for the respective baseline definitions can be as simple as setting confidence intervals [26,70] or as complicated as constructing spatially interpolated maps [29,58]. Commonly used proxies for the characterisation of bioavailable Sr also ranges from modern environmental samples [21,24,26], over soil samples from cultural layers [23,65], modern or archaeological faunal remains [31,33,34], to archaeological human remains [30,31,71]. Each proxy material represents a different part of the Sr isotope cycle and is likely biased towards the dietary Sr isotope composition that was available to ancient humans. We chose to test a variety of proxy archives for the definition of a Sr baseline of the study area in the Prignitz. These are 1) surface water ^87^Sr/^86^Sr signatures, 2) modern soil leachate ^87^Sr/^86^Sr signatures, 3) plant ^87^Sr/^86^Sr signatures, 4) soil ^87^Sr/^86^Sr signatures from the cultural layer of the *Königsgrab* and 5) petrous bone ^87^Sr/^86^Sr signatures of the individuals under investigation. The different bioavailable Sr isotope baselines were calculated as the average ^87^Sr/^86^Sr composition of the respective proxy ± it’s single and double standard deviation and are compared to the underlying proxy ^87^Sr/^86^Sr data in Fig 4.

When considering the baselines calculated using the double standard deviation, these generally return a range wide enough to roughly encompass the majority of underlying baseline data points (Fig 4A), suggesting that chosen statistical approach is suitable. However, depending on the chosen proxy material, the calculated baselines vary drastically, suggesting the presence of proxy-specific biases. Hence, it is vital to consider the potential factors controlling bioavailable ^87^Sr/^86^Sr within the different proxies to determine, which baseline is most likely to reflect dietary ^87^Sr/^86^Sr available to the investigated individuals during the Late Bronze Age. The ^87^Sr/^86^Sr isotope signatures of the modern environmental samples clearly reveal different compositional arrays for the surface waters compared to the plants and soil leachates. This is likely due to surface water ^87^Sr/^86^Sr signatures being influenced by mobile Sr along their entire watershed, while plants and soil leachates only source their bioavailable ^87^Sr/^86^Sr signature from their immediate surrounding. This is reflected in generally similar plant and soil leachate ^87^Sr/^86^Sr signatures, but sometimes drastically different water ^87^Sr/^86^Sr signatures, at the same location in this study (Fig 3) as well as others [22,24,30]. Interestingly, all the baselines based on modern proxies of the study area have an average ^87^Sr/^86^Sr value close to 0.7100, which fits within modelled bioavailable ^87^Sr/^86^Sr ranges for the North German Basin from previous studies [58,61,67], and particularly with the average ^87^Sr/^86^Sr signature of 0.7104 ± 0.0018 calculated with data from [67] for the Elbe River, which dominates the North German Basin. However, the soil leachate and plant baselines defined herein differ from that of the surface waters in that they are around three times wider, mainly due to a wider spread in soil leachate and plant ^87^Sr/^86^Sr values. In this respect, the soil-leachate and plant baseline data presented herein resemble those recently published for Denmark [30], where the surface geological features are characterised by a similar glaciogenic landscape and soils developed on glaciogenic sediments. Besides the study from Denmark, another study from Great Britain also observed significantly more radiogenic soil-leachate and plant ^87^Sr/^86^Sr values compared to their water ^87^Sr/^86^Sr baseline for samples from forested sites [36]. Such discrepancies have been attributed to recent leaching of the natural soil carbonate content due to the natural acidification of forest soils as well as continued carbonate leaching from glacial soils during glacial retreat [30,36]. This could explain the elevated soil leachate and plant ^87^Sr/^86^Sr signatures observed for many of the sampled sites in the Prignitz, such as S08 and S20, suggesting that these represent continuous leaching of originally present carbonate constituents in the glaciogenic tills. Hence, as also suggested for Denmark, the soils of the Prignitz today likely do not reflect their bioavailable strontium isotope composition of prehistoric times. However, several baseline sites also recorded soil leachate and plant ^87^Sr/^86^Sr signatures significantly lower than the corresponding water sample, such as S14 and S21. Since these particular sites are located within close proximity to agricultural lands, it is possible that modern anthropological contamination, such as the use of fertiliser, might explain the offset between the respective proxy ^87^Sr/^86^Sr values. For sites S14 and S21 mentioned above, an anthropological source for the low soil leachate and plant ^87^Sr/^86^Sr values is further supported by their comparatively high soil leachate and plant Sr concentrations. The corresponding water samples do not show significantly higher Sr concentrations or lower ^87^Sr/^86^Sr signatures compared to the other sampled sites, suggesting that the water proxy might be more robust towards local surface soil alterations, such as enhanced acid leaching in forests over time and more recent anthropogenic contamination. Alternatively, the wider spread in ^87^Sr/^86^Sr data of soil leachates and plants could simply be a result of the point sample nature of these sample types versus the averaged-out watershed catchment signatures of surface water samples. However, the ^87^Sr/^86^Sr signature of an individual is always an averaged signature of their food intake and the plant and soil leachate ^87^Sr/^86^Sr signatures representing plant-based food, are fairly evenly distributed, rather than being biased to either a more radiogenic or unradiogenic signature compared to the water samples. Hence, food sourced from the study area is unlikely to reflect the extreme plant and soil leachate ^87^Sr/^86^Sr values, but rather their average, which aligns well with the surface water ^87^Sr/^86^Sr average/baseline. Considering the above, we, therefore, consider the bioavailable Sr isotope baseline based on the water ^87^Sr/^86^Sr signatures the most suitable approximation of bioavailable ^87^Sr/^86^Sr signatures of dietary ^87^Sr/^86^Sr available to the Nordic Bronze Age population of the Prignitz.

The baseline defined by the archaeological soil sample leachates from the cultural layer of the *Königsgrab* is characterised by an average ^87^Sr/^86^Sr signature of 0.7106 with a tight range. It fits well within the ^87^Sr/^86^Sr ranges expected for the North German Basin, and in particular agrees with the water baseline defined for the study area herein (Fig 4B). This suggests that the buried soil underneath the grave as well as the clay floor inside the grave chamber were likely unaffected by recent agricultural contamination and leaching over time, suggesting that archaeological soils might be a suitable proxy for reconstructing prehistoric ^87^Sr/^86^Sr baselines. In comparison, the ^87^Sr/^86^Sr signature of the exposed, modern soil of site S07, less than 200m away from the *Königsgrab*, not only revealed a lower leachate ^87^Sr/^86^Sr signature than its respective water ^87^Sr/^86^Sr value, but also lower than the soils from the *Königsgrab*. Hence, taking deeper soil samples from the cultural layers of Bronze Age excavation sites could be the key to creating reliable and relevant bioavailable Sr isotope baseline suitable for provenance studies in agricultural regions and regions dominated by glaciogenic sediments. However, it is important to note that the homogenous ^87^Sr/^86^Sr signatures measured for the different archaeological soils are likely mainly due to all the samples being taken from the same grave mound and within meters of each other. Hence, more soil samples from the cultural layer of the different Bronze Age excavation sites are necessary to test their robustness against modern and long-term alterations and their suitability as a ^87^Sr/^86^Sr baseline proxy.

Finally, the Sr isotope baseline based on the petrous bone ^87^Sr/^86^Sr signatures of the individuals under investigation revealed a drastically different average ^87^Sr/^86^Sr value (0.7136) than the modern and archaeological environmental proxy baselines. While the environmental proxy baselines all indicate an average dietary ^87^Sr/^86^Sr value of ~0.7100 as local, the calculated petrous bone baseline does not include this value, but falls completely above. This does not fit with modelled bioavailable ^87^Sr/^86^Sr ranges for this region as well as bioavailable ^87^Sr/^86^Sr ranges recorded for geologically similar terrains, such as Denmark [25,30,58,61]. Considering the direct link between the bioavailable Sr isotope composition of the human skeleton to its surroundings, the use of archaeological human remains to determine a baseline appears logical. However, this only works if a high enough number of individuals representative of the larger population dynamic of the area is analysed so that the resulting baseline is not overly biased by the presence of anomalous (non-local) individuals. Most of the investigated petrous bones in this study come from burial mounds, some of which were richly furbished (Supplement). Hence, the individuals they represent are unlikely to correspond to the average local population, but are likely biased towards the upper class.

Due to raised concerns of natural carbonate leaching and anthropogenic contamination in regards to the modern plant and soil leachate proxies as well as an unrepresentative sample distribution and selection in case of the archaeological proxies, we deem the modern soil and plant baselines as well as the archaeological soil and human baselines less suitable than that of the water samples for characterising the bioavailable ^87^Sr/^86^Sr signatures available to ancient individuals living in the Prignitz. Hence, the following interpretations on Bronze Age human mobility using Sr isotopes are largely based on the comparison with the water baseline of ^87^Sr/^86^Sr = 0.7100 ± 0.0017 (Fig 4B).

### Human mobility

#### Constraining the number of investigated individuals.

**Fig 5:**
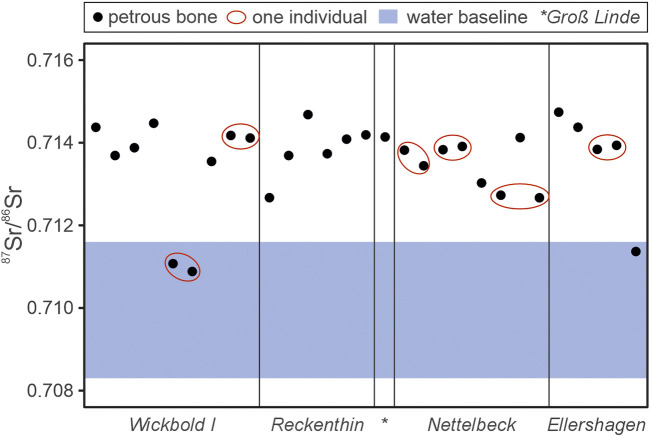
^87^Sr/^86^Sr signatures of the investigated petrous bones plotted over the water Sr isotope baseline.

When comparing the petrous bone ^87^Sr/^86^Sr signatures to the Sr isotope baseline of the surface water from the Prignitz, a surprisingly large number of samples fall outside, suggesting a foreign origin of the respective individuals (Fig 5). However, as indicated by the anthropological investigations, the number of petrous bone samples does not translate 1:1 to the number of individuals. To properly determine the number of foreign individuals in our dataset, the actual number of investigated individuals needs to be constrained first.

As the investigated petrous bones could generally not be assigned to a specific individual in graves including more than one individual, it is possible that some individuals were represented in this study by both of their petrous bones, as also suggested by some of the anthropological investigations (Table 1). Considering that an individual’s two petrous bones form within the same time period, they should source their incorporated Sr from the same pool and, thus, have comparable Sr concentrations and isotope signatures. This is generally confirmed by the Sr data of the petrous bone pairs suggested to be from one individual anthropologically, as most of these pairs returned very similar [Sr] and ^87^Sr/^86^Sr signatures. This suggests that petrous bone Sr data might be an important tool to help in constraining the number of individuals in cremation burials.

When applied to the investigated petrous bones, KF2380-C1 from *Nettelbeck* stands out, as it returned significantly different [Sr] and ^87^Sr/^86^Sr signatures than its supposed counterpart suggested from the anthropological findings. This discrepancy might indicate that there were three instead of only two individuals in this burial. The remaining three samples from the burial all have comparable ^87^Sr/^86^Sr signatures, however, KF2380-B and KF2380-C2 have almost identical [Sr] and ^87^Sr/^86^Sr signatures and are, therefore, the most likely pair from one individual. From *Reckenthin*, KF2376-A and KF2376-B are also suggested to be from one individual anthropologically. The samples have similar ^87^Sr/^86^Sr signatures, but significantly different [Sr], the latter suggesting that the petrous bones might be from different individuals. As the difference in Sr concentration is still reasonable, considering that the determination of Sr concentrations in petrous bones depends strongly on the weighing precisions of the individual otic capsule fractions (estimated to be around ± 10% at sample weights of 1–2 mg), together with the anthropological evidence we propose that samples KF2376-A and KF2376-B from Reckenthin are from one subadult individual.

In addition to verifying whether a pair of petrous bone originates from the same individual based on anthropological investigations, Sr data can serve as a supplementary indicator to identify which petrous bones belong to a single individual in burials containing multiple petrous bones, such as *Wickbold I*. The three possible petrous bone fragments, KF2099-A - KF2099-C, are supposed to correspond to only two individuals, one adult and one subadult. The [Sr] and ^87^Sr/^86^Sr signatures of KF2099-B and KF-2099-C are very similar, potentially indicating that these two petrous bone fragments are from the same individuals. The [Sr] and ^87^Sr/^86^Sr signature of KF2099-A, on the other hand, are very similar to those of KF2093, which corresponds to one of the adult individuals found in the same complex. Hence, these petrous bones could potentially be from one single male adult individual on the basis of their Sr properties, however, the remaining bone fragments associated with KF2099-A appear too small to belong to an adult individual. Therefore, we interpret KF2093 and KF2099-A as two different individuals, while KF2099-B and KF-2099-C represent the same individual, suggesting that the petrous bone samples from complex A represent five individuals in total.

Through combining the anthropological investigations with the Sr results, the total number of investigated individuals can be constrained to 22 individuals, seven from *Wickbold I*, five from *Reckenthin*, one from *Groß Linde*, five from *Nettelbeck* and four from E*llershagen* (Fig 5). However, it is important to note, that, while starkly different petrous bone Sr data are a clear indicator for multiple individuals, very similar Sr results are not necessarily corresponding to one individual, as different individuals with similar diets could also have similar Sr signatures in their petrous bones. Hence, a combination of anthropological investigations and Sr isotope/concentration data is probably the best approach to determine the number of individuals in cremated burials.

#### A Sr isotope perspective.

Of the 22 investigated individuals, only two fall within the local-bioavailable Sr isotope baseline defined by the surface water samples (Fig 5). One of them is a subadult individual from *Wickbold I* (KF2099-B & KF2099-C) and the other is a juvenile to adult female excavated at *Ellershagen* (KF2384). While these two individuals technically could have also originated from another region with a similar or overlapping bioavailable Sr isotope baseline, for example mainland Denmark [25,30], their petrous bone ^87^Sr/^86^Sr signature is interpreted to indicate a local origin within the Prignitz region. The remaining 20 individuals all record petrous bone ^87^Sr/^86^Sr signatures significantly higher than the local baseline and, thus, these individuals likely originated elsewhere. Considering that the investigated surface waters used to establish the local baseline have comparatively low Sr concentrations (Table 2), it is reasonable to suggest that their isotope signature could have been overwritten by other Sr sources, such as the plant-based food, requiring the use of a different baseline. However, when looking at the plant and soil leachate ^87^Sr/^86^Sr data used to define their respective baseline (Fig 4A), the soil leachate ^87^Sr/^86^Sr signatures are consistently too low to explain the ^87^Sr/^86^Sr signatures of the investigated individuals, while the only plant ^87^Sr/^86^Sr signature high enough to explain the petrous bone data revealed a comparatively low Sr concentration. As discussed above, the complete plant and soil leachate data set supports that dietary ^87^Sr/^86^Sr sourced from the study area was likely averaged out to a value close to average surface water ^87^Sr/^86^Sr composition, supporting the application of the surface water baseline and the interpretation that 20 of the 22 investigated individuals likely migrated to Seddin from somewhere else.

Archaeologically, the Prignitz and the area of Seddin, in particular, have been suggested to be at the core of an established long-distance trade network reaching from present-day Italy to present-day Denmark during the 9^th^-8^th^ century BCE [72]. Still, the high individual mobility, as suggested by the Sr results presented in this study, is seemingly overwhelming and the presence of such a large number of non-local individuals is surprising, especially when considering *Wickbold I’s* long period of use as indicated by the ^14^C-dates. One possible explanation for the surprisingly high share of potentially non-local individuals is that the samples investigated in this study might belong to ceremonial graves for individuals who were not part of the local population or that simply did not grow up in the area. The lack of aDNA data due to the crematory practice greatly affects the possibility to shed further light on this. Several questions remain open, for example, were those individual migrants from distant regions or were they part of a local population which for some reason were raised far away from their homeland? Many scenarios are possible to explain the data and due to the limited availability of supplementary data sets, it is difficult to assess such questions at the moment.

However, in the following, we aim to provide preliminary considerations regarding possible regions of origins for the identified non-locals based on their Sr isotope signatures. Considering the established trade routes from Italy to Denmark across northern Germany [72], some of the investigated individuals likely originated from regions along this trade route. Mainland Denmark, the North German Basin, and the Polish Lowlands are generally characterised by bioavailable ^87^Sr/^86^Sr values similar to the surface water baseline of the study area [25,30,58,67,73], suggesting that the non-local individuals did not come from these areas. Italy, on the other hand, is characterised by very heterogenous bioavailable ^87^Sr/^86^Sr distributions with ^87^Sr/^86^Sr signatures similar to those of the investigated foreign individuals in its mountainous regions, particularly north of the Po valley [26,64,74]. Hence, a northward migration of individuals to Seddin is one possibility. However, to explain the elevated ^87^Sr/^86^Sr signals of the investigated non-local individuals with respect to potential regions of origin, it is not necessary to go as far south as Italy, as such radiogenic values can already be found in the Harz mountains, Erzgebirge/Fichtelgebirge, Lusatia or Bohemia [58,67], offering possible regions of origin in central Germany, southwestern Poland or western Czechia. Of these, Bohemia appears to be the most obvious choice considering the good connectivity to the study area through the Elbe river. However, it is important to note that the different individuals identified as non-locals do not record homogeneous ^87^Sr/^86^Sr signatures either, but instead show a range in ^87^Sr/^86^Sr from 0.7127–0.7147, suggesting that the individuals might have originated from more than one region.

Studies focusing on mobility during the first half of the first millennium BCE or more specifically during the Bronze Age Periods IV-V are limited both in number and scope. A pilot study conducted on the Late Bronze Age community burying its dead at the cemetery of Simris II in southeastern Scania, Sweden, identified the migration of individuals to this site, but to a lower degree than unveiled by this study [75]. Similar to *Wickbold I*, the Simris II cemetery revealed the use of house urns as containers for the cremated remains of the deceased, however, one of the two individuals buried in the house urns from Simris II recorded a Sr isotope signature compatible with the local baseline suggesting that the practice might be considered an indicator of long-distance contacts at a community or kin level rather than individually. Another Sr isotope investigation on the roughly contemporary burials at the cemetery at Vollmarshausen, Hessen, Germany, provided little evidence for individual mobility [76], while a study from Denmark discovered two non-locals under eight Period IV-V individuals from Stenildgård on the Jutland Peninsula [77]. Hence, while our understanding of individual mobility in northern Europe during the Bronze Age Periods IV-V is still limited, it has previously been identified, but not to the extent revealed by the case study at Seddin, emphasizing the uniqueness of this Bronze Age communities’ elite.

Mobility data by means of strontium isotope analyses are more abundant when it comes to the late 3rd and the 2nd millennium BCE both in northern Europe [78,79], and in central Europe including northern Italy [74,80]. A study focusing on Final Neolithic and Early Bronze age communities in the Lech valley, Bavaria, to investigate the role of individual mobility as a key factor for the spread of technologies and cultural practices revealed a high female mobility, suggesting that patrilocal residential rules and female exogamy determined individual mobility [80]. While the determined sex in this study on Seddin could not be linked directly to the investigated petrous bones in burials with more than one individual, the single urn burials reveal no such sex-bias for Seddin, as both male and female individuals were identified as non-local. Another study from Germany investigated human remains recovered from the late 2^nd^ millennium battlefield in the Tollense Valley in northeast Germany [79]. While this investigation revealed a high number of male non-locals, the specific archaeological context (a battlefield site) makes the dramatic gender bias rather unsurprising, just as the observations that the strontium isotopic composition of the investigated individuals suggest that the non-locals originated from many different places [79]. Early Nordic Bronze Age mobility has also been investigated within the region of present-day Denmark, where 88 individuals dating from the Neolithic to the Bronze Age were investigated for their Sr isotope composition [78]. Here the number of individuals that likely originated from regions outside present-day Denmark, were only about 20–30%. However, similar to Seddin, the study identified both male and female individuals as non-local, and the diversity of human ^87^Sr/^86^Sr signatures also support that the non-local individuals originated from a variety of places [78]. Looking at northern Italy, Sr isotopes analyses have identified non-locals within the Italian Final Bronze Age community at Frattesina [74] showing a dominant female mobility. Combined, the discussed studies show different forms of mobility and migration took place in and from different regions of Europe throughout the Bronze Age. However, there are still large regional gaps in relation to the coverage of strontium isotope studies, which need to be filled to provide a comprehensive understanding of individual Bronze Age mobility. Finally, while the Seddin individuals reveal an unusually high percentage of non-locals, previous Sr isotope studies suggest a higher degree of mobility among elite population. This supports that the overly high percentage observed in this study is likely due to an underrepresentation of the general population, revealing a clear knowledge gap in understanding Bronze Age mobility at Seddin due to a lack of available human remains from non-elite burials.

#### An archaeology perspective.

Identifying non-local individuals based on archaeological evidence is extremely difficult. Foreign objects, raw materials, motives and decorative influences serve as indicators for exchange practices and potential mobility [78,81–83], however it is generally impossible to tell whether the objects and ideas travelled together with the buried individuals or were brought in by traders, migrants, travellers, visitors or any other possible category of mobile people. It is also very difficult to say how far people moved based solely on the archaeological context. On the other hand, the archaeological context provides a unique source of information helping to identify exchange patterns and routes, thus significantly contributing to the evaluation of the mobility patterns unveiled by the Sr data.

The close spatial and temporal relation of *Wickbold I* and the *Königsgrab* as well as the many similarities with respect to architecture and furnishing suggests a possible close relation between the buried individuals from the two mounds. It is not possible to determine the number, age or biological sex of the buried individuals at the *Wickbold I* mound, however, just like the *Königsgrab*, it definitely included a sword-bearer from period V, likely of high rank. The radiocarbon dates from *Wickbold I* suggest that the mound included several other graves from different periods, some predating its construction. As the Sr data show that most of the individuals irrespective of their chronological characterisation, were of non-local origin, it appears that high mobility characterised the users of the *Wickbold I* burials over a longer time span than Period V. This supports earlier interpretations of this area (and potentially this mound) as bearing a special, maybe ritual, significance [84–86]. On the other hand, as the grave goods recovered in both *Wickbold I* and the *Königsgrab* suggest that the area was well-integrated into both short- and long-distance LBA exchange networks [1,2,13,15,42,45,46,87,88], the presence of non-locals among the sampled individuals should not surprise. One could suggest the possible origin of those individuals by looking at grave goods, however, the grave assemblages from the *Königsgrab* and *Wickbold I* point, as discussed, to both southern and northern influences [2,11] and could have reached the Prignitz as exchanged gifts [15], making them rather unsuitable for tracing their owner’s origin. In general, LBA archaeological assemblages found in the Prignitz suggest close contacts with the southern region of the Nordic Bronze Age (Southern Scandinavia) and indeed some of the individual’s Sr signatures could point to areas on Bornholm or in southern Sweden [75,89]. The archaeological material also suggests exchanges southwards [15,45,46], and a strong relation to the Saale estuary region [13,90]. The closest southern region with documented bioavailable Sr isotope signatures high enough to explain the ^87^Sr/^86^Sr signatures of the *Wickbold I* individuals are, however, west of the Saale, in the Harz mountains and in the Erzgebirge/Fichtelgebirge. It remains unquestionable that the size of the *Königsgrab* burial mound stands out and must have required a considerable investment of resources and work. Despite *Wickbold I’s* smaller size, both mounds appear to be a sort of heroic tombs in Homeric terms with parallels in the contemporary Mediterranean basin [2,91,92].

The investigated individuals from *Reckenthin, Nettelbeck* and *Ellershagen* were also excavated from grave mound burials, although of a lesser monumental nature, while the individual from *Groß Linde* is reported as coming from a single urn find, which could have been part of an urnfield cemetery. In terms of funerary ritual, type of grave and grave goods, all four sites fit well within the expected LBA cultural patterns of the area [46,90,93]. The only exceptions are marked by two items: 1) a ribbed needle with a small head from grave 2 at *Nettelbeck*, which has been associated with similar items from southern Germany [93] and 2) a Lusatian-style *Terrine* from the grave 2 of *Reckenthin* [94]. As discussed before, the foreign character of selected grave goods is no proof of direct contact with the associated regions, however, as in the case of *Wickbold I* and of the *Königsgrab*, the areas suggested by those foreign items fit rather well with some of the non-local Sr signatures obtained in this study.

All in all, there seems to be, on the one hand, a contrast between the strong mobility aspect of the investigated individuals and the apparent local contextual archaeological burial tradition, while, on the other hand, the foreign grave goods point to potential areas of origin that commonly match the non-local Sr signatures. Future systematic analyses on a larger number of contexts are needed to confirm this potential relation.

## Conclusions

This study takes a first look at individual mobility in LBA Seddin by utilising the cremated petrous bone and environmental ^87^Sr/^86^Sr signatures. The main results of this study can be summarized as follows:

1] Choosing the most suitable proxy material to define a meaningful bioavailable Sr isotope baseline for Bronze Age mobility studies in the area around Seddin has proven to be challenging. The modern soil and plant ^87^Sr/^86^Sr signatures are likely altered due to natural carbonate leaching processes over time and/or modern agricultural contamination. Hence, these samples do not seem to adequately reflect the environmental bioavailable Sr isotope signatures that prevailed during the Bronze Age. The archaeological soil samples from the cultural layer of the *Königsgrab* are seemingly not affected by these alteration processes, but are spatially not representative, while the Sr isotope baseline defined by the investigated individuals themselves reveals a strong bias towards the Sr isotope range expected for the local geology, suggesting an over-representation of non-local individuals. Finally, the modern surface water samples appear to be more robust against recent alterations and are further compatible with the soil leachates from the cultural layer. Consequently, the Sr isotope baseline defined by the surface waters is thought to be the best representation of local bioavailable Sr present during the Late Bronze Age and was, therefore, chosen to discriminate between local and non-local individuals studied herein.
2] Our study shows that incorporating petrous bone ^87^Sr/^86^Sr signatures and Sr concentrations alongside anthropological investigations can more accurately constrain the number of investigated individuals found in cremated burials, as petrous bone pairs assigned to the same individual typically exhibit similar ^87^Sr/^86^Sr signatures and Sr concentrations.
3] Of the suggested 22 investigated individuals from Seddin, only two fall within the local Sr isotope baseline established for the study area in the Prignitz, suggesting high individual mobility during the LBA. However, considering that the available bioavailable Sr isotope data off the study area is currently limited to the data in this study, the interpretations on individual mobility made herein could change once more baseline data of the region is available.
4] The 20 individuals identified as non-local all have higher petrous bone ^87^Sr/^86^Sr signatures than the ^87^Sr/^86^Sr baseline of the study area. In northern Germany, such high Sr isotope values have only been recorded in the Harz mountains and Erzgebirge/Fichtelgebirge thus far. However, considering the characteristics of the archaeological record from the region, one cannot rule out the possibility that these individuals came to the Prignitz from more distant regions such as Lusatia, Bohemia, northern Italy, southern Sweden or the Danish Island of Bornholm, as these regions are also characterised with bioavailable Sr isotope baselines that match the non-local petrous bone Sr isotope signatures.
5] The archaeological contexts of the sampled individuals certainly attest to the existence of a powerful elite in the Seddin region with long-distance connections across the continent. However, generally, the burial also shows typical traits for the region, while the grave goods with foreign properties could be imported or manufactured locally according to a foreign style.
6] The overall characteristics of the chosen contexts would speak in favour of a mobile society, but the considerable share of individuals brought up in distant regions is so high that a specific explanatory model needs to be found. Further, it is important to note that the investigated number of individuals thus far is still limited and largely constrained to richer burials which likely results in an interpretation biased by an overrepresentation of the local elite. Hence, a systematic analysis of a larger number of contexts and individuals is needed to properly understand individual mobility patterns in the Prignitz during the Bronze Age.

## Supporting information

S1 Fig Overview for the burial mounds Königsgrab and Wickbold I. A) Digital terrain model of the central landscape of Seddin with the burial mounds Königsgrab and Wickbold I. B) Terrain map of the burial mound Wickbold I with 10 cm isolines and the area of the 2017–2018 excavations outlined in red. C) Metal grave goods from the stone burial chamber of Wickbold I [11]. D) Finds from the stone burial chamber of the Königsgrab [3].
(TIF)

S2 Fig Drone photo of the area excavated in 2017–2018 showing the exposed remains of stone rings and pavements. (TIF)

S3 Fig Plan of the excavation area at the burial mound Wickbold I (1888) investigated in 2017–2018. The different uncovered find concentrations (FC) and structures (S) are outlined with special emphasis given to the find locations of the samples used for Sr isotope analysis (KF) and C radiocarbon dating (UC).
(TIF)

S4 File Supplementary archaeological context Archaeological context for ‘A foreign elite? Identifying mobility patterns at Seddin, Germany, during the Late Bronze Age.’.
(DOCX)
