# Supplementary material for: A Late Bronze Age foreign elite? Investigating mobility patterns at Seddin, Germany
Source: PLoS One. 2025 Sep 10;20(9):e0330390. doi: 10.1371/journal.pone.0330390 (PMC12422465; doi:10.1371/journal.pone.0330390)
Supplement: S4 File — Archaeological context for ‘A foreign elite? Identifying mobility patterns at Seddin, Germany, during the Late Bronze Age.’. (DOCX) [file pone.0330390.s004.docx]

**S4: Supplementary archaeological context for ‘A foreign elite? Identifying mobility patterns at Seddin, Germany, during the Late Bronze Age.’**

1. Newest findings from *Wickbold I*

As part of the excellence cluster TOPOI "The Formation and Transformation of Space and Knowledge in Ancient Civilizations, Research Group B-2: XXL Monumentalized Knowledge. Extra-large Projects in Ancient Civilizations", several archaeological excavations were carried out between 2013 and 2018. The focus of these excavations were two grave mounds located in the Prignitz in the north-western Brandenburg, Germany, the so called *Königsgrab* of Seddin and the *Wickbold I* burial in the forest *Wickboldsches Wäldchen* (Figure S1). Even though the *Königsgrab* and the *Wickbold I* burial mound are located within 1 km of each other, date both to the 9^th^ century BCE (Period V) and were discovered within eleven years of each other, the latter has received significantly less attention in the public eye and scientific community. This is also evident in the various studies published on the newest findings on the *Königsgrab* since the modern excavations (1–7). The *Wickbold I* investigations, on the other hand, are still unpublished.

As the exact location of *Wickbold I* was not documented, its presumed location was determined through literature review and a survey conducted in 2006 (8). The burial chamber, which was removed after its first discovery in 1888, was presumed to be located in the eastern part of the tumulus (9). Hence, the south-eastern quarter of the burial was excavated in 2017 and 2018, uncovering an area of ca. 145 m² (Figures S2- S3). A preliminary report of the modern excavations is given below.

In the centre of the excavated area, a ca. one-meter high vertical profile was exposed (Figure S3). In the north-eastern segment, three to four, in some places even five layers were uncovered. The number of unearthed layers decreased to three or less in the south-western segment, with only one layer in the southern part up to the enclosing stone ring. In the documentation of the site, a distinction was made between original features, which date back to the Bronze Age, and altered features, which were created in 1888 by redeposition. The term ‘find concentration’ (FC) is used for an assembly of archaeological finds without visible boundaries. If a clear interpretation of the excavated features was possible, they are referred to as structures (S). The boundary between original and altered features is roughly layer 3, which, at its highest point in the centre of the excavation area, lays approximately 1m below ground level. The sand above it was mixed with sods in varying orientation and small stones due to being redeposited in 1888.

- 1. Original features and finds

The oldest original features are remains of cremation burials (Figure S3). Some of these features were almost completely destroyed by the activities in the 19th century, such as grave FC8+S2. Due to time constraints, not all features could be fully excavated (e.g. urn grave S4). These features were preserved in situ and should be studied in the future. Based on the available radiocarbon dates UCIAM 245877 and 245875, the original find concentrations FC13 + S5 and FC8 + S2 (Fig. 4) date to the older Bronze Age (Period II-III). Such early dating was not expected and renders these FC older than the previously known graves in the forest *Wickboldsches Wäldchen*. It is not clear whether these features were designed as part of the mound from the beginning or whether they are older burials that were mounded over (on purpose or by accident) at a later stage. The latter has been confirmed for the Ellershagen burial site (10). Radiocarbon dates UCIAM 245873, 246561, 246563 and 246564 from the find concentrations 2, 10 and 14 extend the usage period into Period IV and thus into the Late Bronze Age. Older graves from Period III and IV were also found under the famous Period V grave mound, *Lusehøj*, located on Fyn, Denmark (11)

The next youngest original features consist of stones. At the outer edge of the excavation area, the remains of a ring of larger stones was identified, referred to as the 1^st^ stone ring (Figure S2 - S3). It has a diameter of about 24.60 m. Right outside the 1^st^ stone ring is a pavement consisting of very small and densely set stones, which slopes down and whose boundaries could not be determined. Approximately 6 m inside the 1^st^ stone ring follows the 2^nd^ stone ring, after about 1 more metre the 3^rd^ stone ring and, finally, at an even smaller distance the 4^th^ stone ring, which is not perfectly concentric with the 2^nd^ and 3^rd^ ring. The ground between the 2^nd^ and 3^rd^ stone ring and the 3^rd^ and 4^th^ stone ring is paved with smaller stones. The central structure of rings and pavement is to be regarded as a unit. It is on the level of layer 3 and was partially affected by the activities around 1888. The urn grave S4 lies under the 2^nd^ stone ring, whose position can be reconstructed well suggesting that the stone ring is younger than the grave, which probably belongs to the group of original features of periods III-IV. However, the exact dates of the construction of the stone rings 1-4 could not be determined. Stone rings as boundaries of mounds or as internal constructions are not unusual for the region. In the Prignitz they are associated with high-ranking burials (periods III and IV) around Weitgendorf (12). Stone pavement between stone rings are rarer, but have previously been observed in northeast Germany, e.g. in Zarrenthin, Vorpommern- Greifswald (13), and Buchholz, Uckermark (14). Further, a double stone ring and pavement feature is known from the *Teufelsberg* cemetery near Wolfshagen, Prignitz. It dates to Period V-VI, probably served to enclose ustrines, but was not mounded over (15). However, the best parallel for the multiple stone rings with pavement of *Wickbold I* is the famous *Borum Eshøj* mound in East Jutland, Denmark, whose oak coffin burials were dendrochronologically dated after 1348 BCE- 10/+18 and after 1344 BCE - 11/+18 (16).

- 1. Features altered in 1888

During the discovery and first investigation of *Wickbold I* in 1888, some features of the grave were altered and new ones created. Alternate cuts were probably made to extract stones from the mound. Massive accumulations of cremated remains indicate that their clay vessels were emptied in search of metal grave goods. Considering the high degree of fragmentation of the pottery, the vessels were likely smashed. On the north-eastern edge of the excavation area, FC 10 and 14 lie above the stone pavement outside the 1^st^ stone ring (Figure S3). However, considering the radiocarbon dates UCIAM 246563, 245876 and 246564, it is very likely that at least the Period II cremations were disposed there in 1888, as well as parts of the Periods III-IV urns.

Complex A (Figure S3, yellow) comprises of several in 1888 artificially created find concentrations, which were encountered during the excavation of the second and third layers above the 2^nd^ to 4^th^ stone ring and to the south of them. The elongated shape could indicate the usage of a trench to unearth stones in 1888, which was later backfilled with material from a neighbouring trench. Pottery fragments were found as low as layer 3 and most fragments were recovered in FC 1 to the north of the profile, at the location of the 1m-profile in the centre of the excavation site and in FC 2. However, some fragments were also found outside of complex A. Based on the type and composition of the recovered material from complex A, it was originally assumed that it consisted of the remains of objects from the burial chamber of the sword grave destroyed in 1888. However, the volume of recovered objects almost certainly exceeds the capacity of the burial chamber. Inter alia complex A included fragments of a door urn. Even though the form is completely different from the urn described in the literature (9), it is undoubtedly the piece from the burial chamber. Considering this, it is likely that complex A includes the contents of the burial chamber or parts thereof. West of the northern part of complex A, a polygonal area within the 3^rd^ and 4^th^ stone ring is completely free of stones and pavement. This is probably where the burial chamber stood (Figure S3). The mound, which was removed or transformed in 1888, was probably erected after the burial chamber was built in period V. Alternatively, it is possible that the burial chamber was sunk into an older burial mound with internal structures.

At the edge of FC 1, numerous tiny fragments of a bowl or cup made of extremely thin sheet bronze were recovered right next to a large single stone (Figure S3, light blue). Such fragile bronze bowls are also known from the *Königsgrab* (17). The large stone might have been the seal of the burial chamber that remained on site. From FC 1 the samples UCIAM 246559 and 246560 delivered radiocarbon dates for cremations from periods IV-V, which chronologically could belong to the burials in the burial chamber. These dates are slightly older than the dates MAMS 21018 and 21017 and KIA 21317 (3) from the buried soils under the mound of the *Königsgrab.* If the dated cremation from FC 1 does indeed include remains from the burial chamber, the elite grave *Wickbold I* could be slightly older the *Königsgrab*, despite some overlap. Most of the objects from FC 1 were likely part of the equipment of the burial chamber. However, matching fragments of vessels and objects were also found in FC 2, suggesting that some of the burial chamber equipment was probably also dumped in the area of FC 2 in 1888. Thus, it is surprising that the radiocarbon dates from FC 2 only date between period II-IV (UCIAM 246562, 245873 and 246561) and not Period V. Accordingly, the contents of older graves also ended up in FC 2. Thus, the exact chronology of the different parts of complex A is unclear. The recovered material comes not only from the period V burial chamber, but also from older graves and maybe even from younger burials. Based on the current findings, it is, thus, not possible to reconstruct the number of burials in the burial chamber or to identify the ‘sword-bearer’ among the recovered remains. However, his urn is likely present in the form of a door-urn.

1. Investigated reference sites around Seddin, Prignitz, Brandenburg
   1. *Reckenthin*, site 1

The site, *Reckenthin*, is located in on a saalian ground moraine approximately 8 km southeast of the *Königsgrab* of Seddin. The burial was originally discovered in 1929 and partially destroyed. Following a another partial removal in 1971, a rescue excavation was conducted (18). The excavation focused on a single grave mound and surrounding cemetery, which date to Period IV, potentially reaching into Period V. The single grave mound is built from sand and a single ring of stones with a diameter of 11 m and a height of about 0.7 m (18). For the cemetery, nine graves were documented, some of which might not be urn burials, but pit graves. Grave 1 is located in the northern centre of the grave mound and was lowered into a pit, which is an unusual primary grave for grave mounds dating to Period IV-V. It includes a *Kegelhalsterrine* covered with a bowl as an urn as well as a bronze awl and loop ring. Grave 2 and 3 were found underneath the stone ring of the mound and were interpreted as subsequent burials (18). The graves 6 to 9 are shallow burials underneath a stone cover. The urns commonly associated with graves 2 to 9 are *Kegelhalsterrinen*, but also a pot and a double cone vessel were used. Additionally, a thin-walled Lusatia-style *Terrine* with vertical grooves as a grave good was found in grave 2 (18). However, no metal objects were recovered from any of these graves. The recovered material and structure of the grave are typical for the area, suggesting a local population, with the exception of the Lusatia-style *Terrine* from grave 2, which supports an influence from the Lusatian culture in the southeast.

- 1. *Groß Linde*, site 17

The site is located in the central Prignitz on the top of a saalian ground moraine approximately 3.8km southwest of the *Königsgrab* of Seddin. In 2003, a single urn was found in the roots of a fallen tree (directly north of multiple grave mounds) and recovered by J. Hildebrandt and R. Vocke. As of now, the find is unpublished. Thus far, only the single urn burial is confirmed, which could have been part of a larger cemetery. The urn is a *Kegelhalsterrine* typical for the region, has a faded profile and contains the unstratified cremated remains of a child. The grave is assumed to date to Period V or VI, but lack of archaeological evidence associated with the grave prohibits constraining the material and ritual structures of the grave site. Similar, little information exists on the grave mounds south of the urn grave, which were dated to Period IV-VI (15).

- 1. *Nettelbeck*, site 4

The site is located at the northern border of the Prignitz on the eastern slope of a saalian ground moraine approximately 18 km north of the *Königsgrab* of Seddin. The burial was discovered in 1976 and was part of a rescue excavation in 1979 preceding a highway construction (19). A total of 13 grave mounds were identified during the excavation, of which only seven were investigated. In 1980, another six grave mounds were identified, but not excavated (19). Hence, only the seven grave mounds that were subject of the rescue excavation in 1979 are described in the literature. The investigated grave mounds have diameters of 6m – 10m and heights between 0.6m and 1.2m. The mounds consist mainly of stone, with the exception of one earthen mound. All of the mounds have a stone ring as their outer border. Inside the mounds are furbished with stones constructions, which hold the urns. As urns serve double conical vessels or *Terrinen* with a cover bowl and miniature *Terrinen*, cups, cans, needles and rings and razor were included as grave goods (19). With the exception of one needle from grave mound 2, which could have been imported from Bavaria or Kärnten, there are no obvious foreign influences. Hence, the material and ritual structure of the graves is typical for the area, suggesting the burial of individuals local to the southern zone of the Nordic Bronze Age (19). The initial anthropological investigation discovered that grave mound 4 and 7 only included one individual, while grave mounds 1 – 3 each held 1 adult male and one adult female (19), which does not match the anthropological (and Sr) results of this study. It was suggested that the men in grave mound 2 and 3 were buried without their heads as a warrior tribute, while the presence of the adult females was interpreted as evidence of the practice of widows following their husbands to the grave (19). The grave mounds were in use between Period V and Period VI and were likely graves for the privileged individuals of the community (19).

- 1. *Ellershagen-Rohlsdorf*, site 7

The *Ellershagen-Rohlsdorf* site is located in the northeast of the Prignitz on the northern slope of a saalian ground moraine approximately 21km northeast of the *Königsgrab* of Seddin. It was first discovered in 1974 and was part of a rescue excavation in 1985 (10). Subject of the excavation was one single grave mound dating to Period IV, which included post-burials from pre-Roman Iron Age. The mound was erected over older simple Bronze Age urn burials dating to Period III. The oldest finds, dating to Period III, are the urn burials 1 – 4 using *Kegelhalsterrine*, profiled *Terrinen* with a conical neck and double conical vessels as urns (10). Grave 1 included additional vessels and grave 2 a double button made out of bone as grave goods. All four graves included the cremated remains of one adult individual, of which the individual of grave 1 was suggested to be female and the individual of grave 2 to be male (10).

The later erected grave mound is characterised by a 8 m wide stone ring framed by a 1 m wide stone pavement (10). Urn grave 5, which is contemporaneous to the grave mound, is nestled in a chest like stone construction. A double conical vessel with a cover bowl, which display some Lusatian influences, served as the urn and held the cremated remains of one middle aged man (10). An open arm ring and a stitching awl made of Bronze as well as two cups with handles and a funnel edge are documented as grave goods. The stitching awl and double conical vessel date grave 5 and, thus, the grave mound to Period IV (10). The remains of another cremated individual as well as the remains of a spiral ring and a bulbous can were recovered from the soil layer above and next to grave 5. Slightly southeast to the centre of the grave mound the remains of a third adult and a cup were found. The original anthropological review suggests that all three of these individuals were male (10), but the anthropological review conducted in this study could only identify one individual as likely female. Overall, the material and ritual structure the Period III and IV finds from *Ellershagen-Rohlsdorf* suggest the burial of a local family-sized community, whereas the single individual from grave 5 appears to have received special emphasis (10).

Approximately 1 m above the Period III and IV finds the crown of the grave mound is formed by an accumulation of larger stones. Within these two post burials, grave 6 and 7, dating to the pre-Roman Iron Age were located. Both include the cremated remains of a single adult individual, of which the individual from grave 6 was suggested to be female (10).

1. Petrous bone samples included in this study
   1. Petrous bones from *Wickold I*

KF2093 (Fundkonzentration2-SK2017:307/27/5), KF2094(Fundkonzentration2-SK2017:307/29/4), KF2095 (Fundkonzentration15-SK2018:324/Tuete38), KF2099-A - KF2099-C (Streufunde_Sk2018:324/Tuete51):

- one left and two right petrous bones as well as three petrous bone fragments
- from complex A
- anthropologically at least five individuals, three likely male adults, one infans II to juvenile of undetermined age and one infans I of undetermined age, were identified

KF2096 (Fundkonzentration14-SK2018:324/Tuete33):

- one pars petros fragment
- from FC 14
- anthropologically one late juvenile to senile individual was identified

KF2097 (Befund4-Sk2018:324/Tuete2) and KF2098 (Befund4-Sk2018:324/Tuete3):

- two petrous bone fragments
- from grave S4
- anthropologically one late juvenile to early adult individual was identified
  1. Petrous bones from *Reckenthin*, site 1

KF2373 (1971:20/1/2):

- one left petrous bone
- from grave 1 located north to the center of the grave mound (potentially the primary burial)
- anthropologically one individual, likely a late juvenile-senile female, was identified
- the urn was a *Kegelhalsterterrine* with a cover bowl and the grave goods included a bronze awl and loop ring
- the burial dates to Period IV or potentially V

KF2374-A and KF2374-B (1971:20/2/2):

- two right petrous bones
- from grave 2 located inside the stone ring
- anthropologically two individuals, the first juvenil, the second infans I and both of undetermined sex, were identified
- the urn was likely a *Kegelhalsterterrine* with a cover bowl and the grave goods included the remains of a Lusatian-style *Terrine*
- the burial dates to Period IV

KF2375 (1971:20/4/2):

- one left petrous bone
- from grave 4
- anthropologically a late juvenile to adult individual of undetermined sex was identified
- the urn was a smooth, tall double conical vessel
- the burial dates to Period IV

KF2376-A and KF 2376-B (1971:20/7/2)

- one left and one right petrous bone
- from grave 7
- anthropologically two individuals, the first perhaps female and late juvenile-adult and the second an infans of undetermined sex, were identified
- the urn was likely a bulbous *Terrine* and the grave goods included three more smaller bulbous *Terrine*
  1. Petrous bones from *Groß Linde*, site 17:

KF2377 (2006:464/3):

- one right petrous bone
- from a single urn grave without any grave goods
- anthropologically an infans I individual of undetermined sex was identified
- the burial dates to Period V or VI
  1. Petrous bones from *Nettelbeck*, site 4

KF 2378-A and KF 2378-B (1979:5/2/6):

- one left and one right petrous bone
- from urn grave in the polygonal grave chamber of grave mound 2
- first anthropological investigations suggested one headless adult male and one adult female, but this study’s anthropological (and Sr) findings support only one potentially female, likely adult to senile
- the grave includes a needle with foreign properties, but also local grave goods
- the burial dates to the end of Period V to the first half of Period VI

KF 2379-A and KF 2379-B (1979:5/3/5):

- one left and one right petrous bone
- from urn grave in the polygonal grave chamber of grave mound 3
- first anthropological investigations suggested one headless adult male and one adult female, but this study’s anthropological (and Sr) findings support only one potentially male, likely adult to mature
- the grave includes a needle imitating the needle from grave mound 2
- the burial dates to Period V

KF 2380-A, KF 2380-B, KF 2380-C1 und KF 2380_C2 (1979:5/4/5):

- one left and one right petrous bone as well as two petrous bone fragments
- from urn grave in the polygonal grave chamber of grave mound 4
- first anthropological investigations suggested one adult female, but this study’s anthropological findings support the presence of two individuals (one late juvenile to senile potentially female and one infans I-II of undetermined sex), while the Sr results suggest the presence of three individuals
- the burial dates to Period V
  1. Petrous bones from *Ellershagen*, site 7:

KF2381 (1985:8/1/2):

- one right petrous bone
- from grave 1
- anthropologically two individuals, one late juvenile to senile potentially female and one infans I of undetermined sex, were identified
- urn grave with four additional vessels as grave goods
- the burial dates to Period III

KF2382 (1985:8/2/2):

- one right petrous bone
- from grave 2
- anthropologically one adult to early mature likely male individual was identified
- urn grave with stone cover and a double button from bone as a grave good
- the burial dates to Period III

KF2383-A and KF2383-B (1985:8/5/2):

- one left and one right petrous bone
- from grave 5 (primary grave of a grave mound with stone ring)
- first anthropological investigations suggested one adult male, but this study’s anthropological findings suggest one late adult to mature potentially female
- a burial dispersed over an urn grave in a chest-like stone construction, the surrounding soil layer another grave with a bronze awl, arm ring and spiral ring as well as multiple vessels as grave goods
- the burial dates to Period IV

KF2384 (1985:8/8/11):

- one left petrous bone
- from grave 6 (post-burial)
- anthropologically one juvenile to adult, potentially female individual was identified
- part of two burials later added to the grave mound with clay vessels as grave goods
- the burial dates to pre-roman iron age

1. References

1. Brunke H, Bukowiecki E, Cancik-Kirschbaum E, Eichmann R, van Ess M, Gass A, et al. Thinking Big: Research in Monumental Constructions in Antiquity. 2016 [cited 2024 Apr 25]; Available from: https://refubium.fu-berlin.de/handle/fub188/21854

2. Hansen S, Schopper F, editors. Der Grabhügel von Seddin im norddeutschen und südskandinavischen Kontext: internationale Konferenz, 16. bis 20. Juni 2014, Brandenburg an der Havel. 1. Auflage. Zossen: Brandenburgisches Landesamt für Denkmalpflege und Archäologisches Landesmuseum, OT Wünsdorf; 2018. 147 p. (Arbeitsberichte zur Bodendenkmalpflege in Brandenburg).

3. May J. Neue Forschung am “Koenigsgrab” von Seddin. In: Der Grabhügel von Seddin im norddeutschen und südskandinavischen Kontext. 1. Auflage. Zossen: Brandenburgisches Landesamt für Denkmalpflege und Archäologisches Landesmuseum, OT Wünsdorf; 2018. p. 9–35. (Arbeitsberichte zur Bodendenkmalpflege in Brandenburg).

4. May J. Fokussieren, Positionieren, Schritthalten. Aspekte von Raum und Zeit am “Königsgrab” von Seddin. In: Das Ganze ist mehr als die Summe seiner Teile Festschrift für Jürgen Kunow anlässlich seines Eintritts in den Ruhestand [Internet]. Bonn: LVR-Amt für Bodendenkmalpflege im Rheinland; 2018 [cited 2024 Apr 25]. p. 405–18. (Materialien zur Bodendenkmalpflege im Rheinland). Available from: https://www.academia.edu/38450506/Fokussieren_Positionieren_Schritthalten_Aspekte_von_Raum_und_Zeit_am_K%C3%B6nigsgrab_von_Seddin

5. Haburaj V, Nykamp M, May J, Hoelzmann P, Schütt B. On-Site VIS-NIR Spectral Reflectance and Colour Measurements - A Fast and Inexpensive Alternative for Delineating Sediment Layers Quantitatively? A Case Study from a Monumental Bronze Age Burial Mound (Seddin, Germany). Heritage. 2020;3:528–48.

6. Nykamp M, Hardt J, Hoelzmann P, May J, Reimann T. Towards timing and stratigraphy of the Bronze Age burial mound royal tomb (Königsgrab) of Seddin (Brandenburg, northeastern Germany). E&amp;G Quaternary Sci J. 2021 Jan 12;70(1):1–17.

7. Nykamp M, Hauschulz S, Hardt J, Knitter D, May J, Hoelzmann P. The landscape of the Late Bronze Age royal tomb of Seddin (NE Germany): linking geomorphology, archaeology, and historic evidence. Journal of Maps. 2022 Jan 11;1–11.

8. May J, Hauptmann T. Zerstört-vergessen-wiederentdeckt. Das Gräberfeld in den Wickboldschen Tannen bei Seddin, Lkr. Prignitz. Archäologie in Berlin und Brandenburg 2008. 2010;49–51.

9. Götze A. Hügelgräber bei Seddin, Kreis West-Priegnitz. In 1894. p. 82–9. (Nachrichten über deutsche Altertumsfunde).

10. Breddin R. Ein jungbronzezeitlicher Grabhügel von Rohlsdorf-Ellershagen, Kr. Pritzwalk. Veröffentlichungen des Museums für Ur- und Frühgeschichte Potsdam. 1990;24:53–61.

11. Thrane H. Southwest Funen in its chronological setting- the best parallel of Seddin? In: Der Grabhügel von Seddin im norddeutschen und südskandinavischen Kontext. 1. Auflage. Zossen: Brandenburgisches Landesamt für Denkmalpflege und Archäologisches Landesmuseum, OT Wünsdorf; 2018. p. 91–104. (Arbeitsberichte zur Bodendenkmalpflege in Brandenburg).

12. Horst F. Das mittelbronzezeitliche Hügelgräberfeld von Weitgendorf, Kr. Pritzwalk. Veröffentlichungen des Museums für Ur- und Frühgeschichte Potsdam. 1987;21:131–44.

13. Forler D. Zarrenthin, Lkr. Demmin. In: Bodendenkmalpflege in Mecklenburg-Vorpommern 2005. Archäologisches Freilichtmuseum Groß Raden; 2006. p. 377–9. (53).

14. Schoknecht U. Ein Hügelgrab von Buchholz, Kreis Templin. In: Bodendenkmalpflege in Mecklenburg, Jahrbuch 1961. Museum für Ur- und Frühgeschichte Schwerin; 1961. p. 179–202.

15. Bohm W. Die Vorgeschichte des Kreises Westprignitz [Internet]. Vol. 11. Leipzig: Curt Kabitzsch; 1937 [cited 2024 May 5]. 200 p. Available from: https://journals.ub.uni-heidelberg.de/index.php/nnu/article/view/70338

16. Frost L, Løvschal M, Lindegaard MR, Holst MK. Borum Eshøj Revisited: Bronze Age monumental burial traditions in eastern Jutland, Denmark. Danish Journal of Archaeology. 2017 May 1;6:31–49.

17. Kiekebusch A. Das Königsgrab von Seddin [Internet]. Augsburg: Benno Filser; 1928 [cited 2024 May 7]. 45 p. Available from: https://www.booklooker.de/B%C3%BCcher/Albert-Kiekebusch+Das-K%C3%B6nigsgrab-von-Seddin-F%C3%BChrer-zur-Urgeschichte-Band-1/id/A00Ul1rt01ZZv

18. Breddin R. Untersuchung eines jungbronzezeitlichen Grabhügels von Reckenthin, Kr. Pritzwalk. Ausgrabungen und Funde. 1973;18(3):129–34.

19. Breddin R. Untersuchungen eines spätbronzezeitlichen Grabhügelfeldes der Seddiner Gruppe bei Nettelbeck, Kr. Pritzwalk. Veröffentlichungen des Museums für Ur- und Frühgeschichte Potsdam. 1983;17:49–72.

20. Sabatini S. Late Bronze Age long distance exchanges, agency, and the house urn from the so-called Wickbold 1 mound. In: Der Grabhügel von Seddin im norddeutschen und südskandinavischen Kontext. 1. Auflage. Zossen: Brandenburgisches Landesamt für Denkmalpflege und Archäologisches Landesmuseum, OT Wünsdorf; 2018. p. 51–64. (Arbeitsberichte zur Bodendenkmalpflege in Brandenburg).

21. May J, Hauptmann T. Das “Königsgrab” von Seddin und sein engeres Umfeld im Spiegel neuer Feldforschungen. In: Gräberlandschaften der Bronzezeit. Darmstadt: Internationales Kolloquium zur Bronzezeit; 2012. p. 77–104. (Bodenaltertümer Westfalens).
